# European species of *Dendrostoma* (Diaporthales)

**DOI:** 10.3897/mycokeys.59.37966

**Published:** 2019-10-16

**Authors:** Walter M. Jaklitsch, Hermann Voglmayr

**Affiliations:** 1 Institute of Forest Entomology, Forest Pathology and Forest Protection, Department of Forest and Soil Sciences, BOKU-University of Natural Resources and Life Sciences, Franz Schwackhöfer Haus, Peter-Jordan-Straße 82/I, 1190 Vienna, Austria University of Natural Resources and Life Sciences Vienna Austria; 2 Division of Systematic and Evolutionary Botany, Department of Botany and Biodiversity Research, University of Vienna, Rennweg 14, 1030 Vienna, Austria University of Vienna Vienna Austria

**Keywords:** *
Amphiporthe
*, *
Cryptodiaporthe
*, multi-gene phylogeny, pyrenomycetes, systematics

## Abstract

European species of the genus *Dendrostoma* (Erythrogloeaceae, Diaporthales) occurring on *Castanea sativa* and *Quercus* spp. based on freshly collected material are presented. Using a matrix of sequences from ITS, LSU, *rpb2*, and *tef1*, five species are recognized, and their phylogenetic positions are determined. Four species are added to the 14 described species of *Dendrostoma*. *Dendrostoma atlanticum* on *Castanea sativa*, *D. creticum* on *Quercus coccifera* and *D. istriacum* on *Q. ilex* are described as new species, *Valsa castanea* is combined in *Dendrostoma*, and *D. leiphaemia* is redescribed and illustrated. A key to the European species of *Dendrostoma* is provided.

## Introduction

The genus *Cryptodiaporthe*, based on *Cryptospora aesculi*, is one of several segregates from the large genus *Diaporthe* (Diaporthales), characterized by the lack of stromatic zones and with asexual morphs recognized by Petrak (1921) as *Septomyxa*. In 1933, Wehmeyer (1933) recognized the relatively large number of species with a simple type of stroma development having various asexual morphs as a heterogeneous grouping. Petrak (1971) removed *C. tiliae* (as *C. hranicensis*) to his new genus *Amphiporthe*, mainly due to its *Amphicytostroma* asexual morph, where subsequently several species were added. Using the phylogenetic markers ITS, LSU, and *rpb2*, Mejía et al. (2008) detected that *C. aesculi* is congeneric with the generic type of *Plagiostoma*, *P. euphorbiae*. Thus, *Cryptodiaporthe* became a synonym of *Plagiostoma*. Subsequently (Mejía et al. 2011), several other species of *Cryptodiaporthe* were combined in *Plagiostoma*. Since the first phylogenetic treatment of the Diaporthales using DNA data (Castlebury et al. 2002), many old genera have been split and new ones described, and the proliferation of family names has forwarded a current number of 28, more than a half of which having been erected during the last three to four years (compare Jaklitsch et al. (2016), who listed 11 families). One of these families is the Erythrogloeaceae, whose members are based on phytopathogenic coelomycetous fungi (*Chrysocrypta*, *Disculoides*, *Erythrogloeum*). The only genus of this family for which sexual morphs are known is *Dendrostoma* (Fan et al. 2018). This genus is characterized by features common to many other diaporthalean genera forming pseudostromata lacking black stromatic margins, including *Amphiporthe* and Plagiostoma (Cryptodiaporthe). Rossman et al. (2015) already noted that *Amphiporthe castanea* and *A. leiphaemia* are not congeneric with *A. tiliae* (syn. *A. hranicensis*) and would need a new generic name. *Amphiporthe leiphaemia* was combined in *Dendrostoma* by Senanayake et al. (2018), based on ITS and LSU sequences of a CBS strain without giving any further information, whereas *A. castanea* has not been treated recently, although Jiang et al. (2019), who substantially enlarged the scope of the genus by describing 10 new species from *Castanea* and *Quercus* in China, recognized seven species on *Castanea mollissima*. Here we report on recently collected species of *Dendrostoma* occurring on *Castanea sativa* and *Quercus* spp. in Europe.

## Materials and methods

### Sample sources

All isolates included in this study originated from ascospores of freshly collected specimens derived from recently dead branches or twigs. Details of the strains including NCBI GenBank accession numbers of gene sequences used to compute the phylogenetic trees are listed in Table 1. Strain acronyms other than those of official culture collections are used here primarily as strain identifiers throughout the work. Representative isolates have been deposited at the Westerdijk Fungal Biodiversity Centre (CBS-KNAW), Utrecht, The Netherlands. Details of the specimens used for morphological investigations are listed in the Taxonomy section under the respective descriptions. Freshly collected specimens have been deposited in the Fungarium of the Department of Botany and Biodiversity Research, University of Vienna (WU).

**Table 1.** Isolates and accession numbers of sequences used in the phylogenetic analyses.

Species	Culture^1,2^	Country	Host	Host family	GenBank accession numbers^2^			
					ITS	LSU	*rpb2*	*tef1*
*Chrysocrypta corymbiae*	CBS 132528*	Australia	*Corymbia* sp.	Myrtaceae	JX069867	JX069851	MH545415	MH545457
*Dendrostoma atlanticum*	**D196** = CBS 145804*	France	*Castanea sativa*	Fagaceae	**MN447223**	**MN447223**	**MN432160**	**MN432167**
	**D303**	Spain	*Castanea sativa*	Fagaceae	**MN447224**	**MN447224**	**MN432161**	**MN432168**
*Dendrostoma aurorae*	CFCC 52753*	China	*Castanea mollissima*	Fagaceae	MH542498	MH542646	MH545405	MH545447
	CFCC 52754	China	*Castanea mollissima*	Fagaceae	MH542499	MH542647	MH545406	MH545448
*Dendrostoma castaneae*	CFCC 52745*	China	*Castanea mollissima*	Fagaceae	MH542488	MH542644	MH545395	MH545437
	CFCC 52746	China	*Castanea mollissima*	Fagaceae	MH542489	–	MH545396	MH545438
	CFCC 52747	China	*Castanea mollissima*	Fagaceae	MH542490	–	MH545397	MH545439
	CFCC 52748	China	*Castanea mollissima*	Fagaceae	MH542491	–	MH545398	MH545440
	CFCC 52749	China	*Castanea mollissima*	Fagaceae	MH542492	MH542645	MH545399	MH545441
	CFCC 52750	China	*Castanea mollissima*	Fagaceae	MH542493	–	MH545400	MH545442
	CFCC 52751	China	*Castanea mollissima*	Fagaceae	MH542494	–	MH545401	MH545443
	CFCC 52752	China	*Castanea mollissima*	Fagaceae	MH542495	–	MH545402	MH545444
*Dendrostoma castaneicola*	CFCC 52743*	China	*Castanea mollissima*	Fagaceae	MH542496	–	MH545403	MH545445
	CFCC 52744	China	*Castanea mollissima*	Fagaceae	MH542497	–	MH545404	MH545446
*Dendrostoma castaneum*	**D192** = CBS 145803	Austria	*Castanea sativa*	Fagaceae	**MN447225**	**MN447225**	**MN432162**	**MN432169**
	**D230**	Italy	*Castanea sativa*	Fagaceae	**MN447226**	**MN447226**	–	**MN432170**
	**D260**	Italy	*Castanea sativa*	Fagaceae	**MN447227**	**MN447227**	–	–
*Dendrostoma chinense*	CFCC 52755*	China	*Castanea mollissima*	Fagaceae	MH542500	MH542648	MH545407	MH545449
	CFCC 52756	China	*Castanea mollissima*	Fagaceae	MH542501	MH542649	MH545408	MH545450
	CFCC 52757	China	*Castanea mollissima*	Fagaceae	MH542502	MH542650	MH545409	MH545451
	CFCC 52758	China	*Castanea mollissima*	Fagaceae	MH542503	MH542651	MH545410	MH545452
*Dendrostoma creticum*	**D124** = CBS 145802*	Greece	*Quercus coccifera*	Fagaceae	**MN447228**	**MN447228**	**MN432163**	**MN432171**
*Dendrostoma dispersum*	CFCC 52730*	China	*Quercus* sp.	Fagaceae	MH542467	MH542629	MH545374	MH545416
	CFCC 52731	China	*Quercus* sp.	Fagaceae	MH542468	MH542630	MH545375	MH545417
*Dendrostoma istriacum*	**D122** = CBS 145801*	Croatia	*Quercus ilex*	Fagaceae	**MN447229**	**MN447229**	**MN432164**	**MN432172**
*Dendrostoma leiphaemia*	**D105** = CBS 145800	Austria	*Quercus robur*	Fagaceae	**MN447230**	**MN447230**	**MN432165**	**MN432173**
	**D144**	Poland	*Quercus robur*	Fagaceae	**MN447231**	**MN447231**	**MN432166**	**MN432174**
	CBS 187.37	NA	*Quercus* sp.	Fagaceae	MH855882	MH867393	–	–
*Dendrostoma mali*	CFCC 52102*	China	*Malus spectabilis*	Rosaceae	MG682072	MG682012	MG682032	MG682052
*Dendrostoma osmanthi*	CFCC 52106*	China	*Osmanthus fragrans*	Oleaceae	MG682073	MG682013	MG682033	MG682053
	CFCC 52107	China	*Osmanthus fragrans*	Oleaceae	MG682075	MG682015	MG682035	MG682055
	CFCC 52108	China	*Osmanthus fragrans*	Oleaceae	MG682074	MG682014	MG682034	MG682054
	CFCC 52109	China	*Osmanthus fragrans*	Oleaceae	MG682076	MG682016	MG682036	MG682056
*Dendrostoma parasiticum*	CFCC 52761	China	*Castanea mollissima*	Fagaceae	MH542480	MH542636	MH545387	MH545429
	CFCC 52762*	China	*Quercus wutaishanica*	Fagaceae	MH542482	MH542638	MH545389	MH545431
	CFCC 52763	China	*Castanea mollissima*	Fagaceae	MH542481	MH542637	MH545388	MH545430
	CFCC 52764	China	*Quercus aliena*	Fagaceae	MH542483	MH542639	MH545390	MH545432
	CFCC 52765	China	*Castanea mollissima*	Fagaceae	MH542484	MH542640	MH545391	MH545433
	CFCC 52766	China	Quercus aliena var. acutiserrata	Fagaceae	MH542485	MH542641	MH545392	MH545434
*Dendrostoma qinlingense*	CFCC 52732*	China	*Quercus wutaishanica*	Fagaceae	MH542471	MH542633	MH545378	MH545420
	CFCC 52733	China	Quercus aliena var. acutiserrata	Fagaceae	MH542472	MH542634	MH545379	MH545421
*Dendrostoma quercinum*	CFCC 52103*	China	*Quercus acutissima*	Fagaceae	MG682077	MG682017	MG682037	MG682057
	CFCC 52104	China	*Quercus acutissima*	Fagaceae	MG682078	MG682018	MG682038	MG682058
	CFCC 52105	China	*Quercus acutissima*	Fagaceae	MG682079	MG682019	MG682039	MG682059
*Dendrostoma quercus*	CFCC 52734	China	*Quercus* sp.	Fagaceae	MH542473	–	MH545380	MH545422
	CFCC 52735	China	*Quercus* sp.	Fagaceae	MH542474	–	MH545381	MH545423
	CFCC 52736	China	*Quercus* sp.	Fagaceae	MH542478	–	MH545385	MH545427
	CFCC 52737	China	*Quercus* sp.	Fagaceae	MH542475	–	MH545382	MH545424
	CFCC 52738	China	*Quercus* sp.	Fagaceae	MH542477	–	MH545384	MH545426
	CFCC 52739*	China	*Quercus* sp.	Fagaceae	MH542476	MH542635	MH545383	MH545425
	CFCC 52740	China	*Quercus* sp.	Fagaceae	MH542479	–	MH545386	MH545428
*Dendrostoma shaanxiense*	CFCC 52741*	China	*Castanea mollissima*	Fagaceae	MH542486	MH542642	MH545393	MH545435
	CFCC 52742	China	*Castanea mollissima*	Fagaceae	MH542487	MH542643	MH545394	MH545436
*Dendrostoma shandongense*	CFCC 52759*	China	*Castanea mollissima*	Fagaceae	MH542504	MH542652	MH545411	MH545453
	CFCC 52760	China	*Castanea mollissima*	Fagaceae	MH542505	MH542653	MH545412	MH545454
*Disculoides eucalypti*	CBS 132183*	Australia	*Eucalyptus* sp.	Myrtaceae	JQ685517	JQ685523	MH545413	MH545455
*Disculoides eucalyptorum*	CBS 132184*	Australia	*Eucalyptus viminalis*	Myrtaceae	JQ685518	JQ685524	MH545414	MH545456

^1^ Ex-type strains marked by an asterisk.; ^2^ Abbreviations: **CBS**: Culture collection of the Westerdijk Fungal Biodiversity Institute, Utrecht, The Netherlands; **CCFC**: China Forestry Culture Collection Centre, Beijing, China; ^3^ Isolates/sequences in bold were isolated/sequenced in the present study.

### Morphology

Microscopic observations were made in tap water except where noted. Morphological analyses of microscopic characters were carried out as described by Jaklitsch (2009). Methods of microscopy included stereomicroscopy using a Nikon SMZ 1500 and Nomarski differential interference contrast (DIC) using the compound microscopes Nikon Eclipse E600 or Zeiss Axio Imager.A1 equipped with a Zeiss Axiocam 506 colour digital camera. Images and data were gathered using a Nikon Coolpix 4500 or a Nikon DS-U2 digital camera and measured by using the NIS-Elements D v. 3.0 or 3.22.15 or Zeiss ZEN Blue Edition software packages. For certain images of ascomata the stacking software Zerene Stacker v. 1.04 (Zerene Systems LLC, Richland, WA, USA) was used. Measurements are reported as maxima and minima in parentheses and the range representing the mean plus and minus the standard deviation of the number of measurements given in parentheses.

### Culture preparation, DNA extraction, PCR, and sequencing

Ascospore isolates were prepared and grown on 2% corn meal dextrose agar (CMD; CMA: Sigma, St Louis, Missouri; supplemented with 2% (w/v) D(+)-glucosemonohydrate) or 2% malt extract agar (MEA; 2% w/v malt extract, 2% w/v agar-agar; Merck, Darmstadt, Germany). Cultures are illustrated in Figure 2. Growth of liquid cultures and extraction of genomic DNA was performed as reported previously (Voglmayr and Jaklitsch 2011; Jaklitsch et al. 2012) using the DNeasy Plant Mini Kit (QIAgen GmbH, Hilden, Germany). The following loci were amplified and sequenced: a ca 1.6 kb fragment containing the terminal part of the small subunit nuclear ribosomal DNA (nSSU rDNA), the complete internal transcribed spacer region (ITS1-5.8S-ITS2) and a ca 900 bp fragment of the large subunit nuclear ribosomal DNA (nLSU rDNA), amplified and sequenced as a single fragment with primers V9G (De Hoog and Gerrits van den Ende 1998) and LR5 (Vilgalys and Hester 1990); a ca 1.2 kb fragment of the RNA polymerase II subunit 2 (*rpb2*) gene with primers fRPB2-5f and fRPB2-7cr (Liu et al. 1999) or dRPB2-5f and dRPB2-7r (Voglmayr et al. 2016); a ca 1.3–1.5 kb fragment of the translation elongation factor 1-alpha (*tef1*) gene with primers EF1-728F (Carbone and Kohn 1999) and TEF1LLErev (Jaklitsch et al. 2005). PCR products were purified using an enzymatic PCR cleanup (Werle et al. 1994) as described in Voglmayr and Jaklitsch (2008). DNA was cycle-sequenced using the ABI PRISM Big Dye Terminator Cycle Sequencing Ready Reaction Kit v. 3.1 (Applied Biosystems, Warrington, UK) and the PCR primers; in addition, primers ITS4 (White et al. 1990), LR2R-A (Voglmayr et al. 2012), and LR3 (Vilgalys and Hester 1990) were used for the SSU-ITS-LSU region, and TEF1_INTF (forward, Jaklitsch 2009) and TEFD_iR1 (reverse, 5’ GAGTTYGAGGCYGGTATCTC 3’) or TEF1_INT2 (Voglmayr and Jaklitsch 2017) for *tef1*. Sequencing was performed on an automated DNA sequencer (3730xl Genetic Analyzer, Applied Biosystems).

### Phylogenetic analyses

The newly generated sequences were aligned with the sequences of Jiang et al. (2019), and a combined matrix of the three loci (partial SSU-ITS-LSU rDNA, *rpb2*, and *tef1*) was produced for phylogenetic analyses, with three species (*Chrysocrypta corymbiae*, *Disculoides eucalypti*, and *Disculoides eucalyptorum*) added as the outgroup according to Jiang et al. (2019). The GenBank accession numbers of sequences used in the analyses are given in Table 1. Sequence alignments were produced with the server version of MAFFT (http://mafft.cbrc.jp/alignment/server/), checked and refined using BioEdit v. 7.2.6 (Hall 1999). The combined data matrix contained 4194 characters, viz. 1637 nucleotides of SSU-ITS-LSU, 1075 nucleotides of *rpb2*, and 1482 nucleotides of *tef1*.

Maximum parsimony (MP) analyses were performed with PAUP v. 4.0a165 (Swofford 2002). All molecular characters were unordered and given equal weight; analyses were performed with gaps treated as missing data; the COLLAPSE command was set to MINBRLEN. MP analysis of the combined multilocus matrix was done using 1000 replicates of heuristic search with random addition of sequences and subsequent TBR branch swapping (MULTREES option in effect, steepest descent option not in effect). Bootstrap analyses with 1000 replicates were performed in the same way but using 10 rounds of random sequence addition and subsequent branch swapping during each bootstrap replicate.

Maximum likelihood (ML) analyses were performed with RAxML (Stamatakis 2006) as implemented in raxmlGUI 1.3 (Silvestro and Michalak 2012), using the ML + rapid bootstrap setting and the GTRGAMMA substitution model with 1000 bootstrap replicates. The matrix was partitioned for the different gene regions. In the Results and Discussion, bootstrap values below 70% are considered low, between 70–90% medium, and above 90% high.

## Results

### Phylogenetic analyses

**Figure 1.**
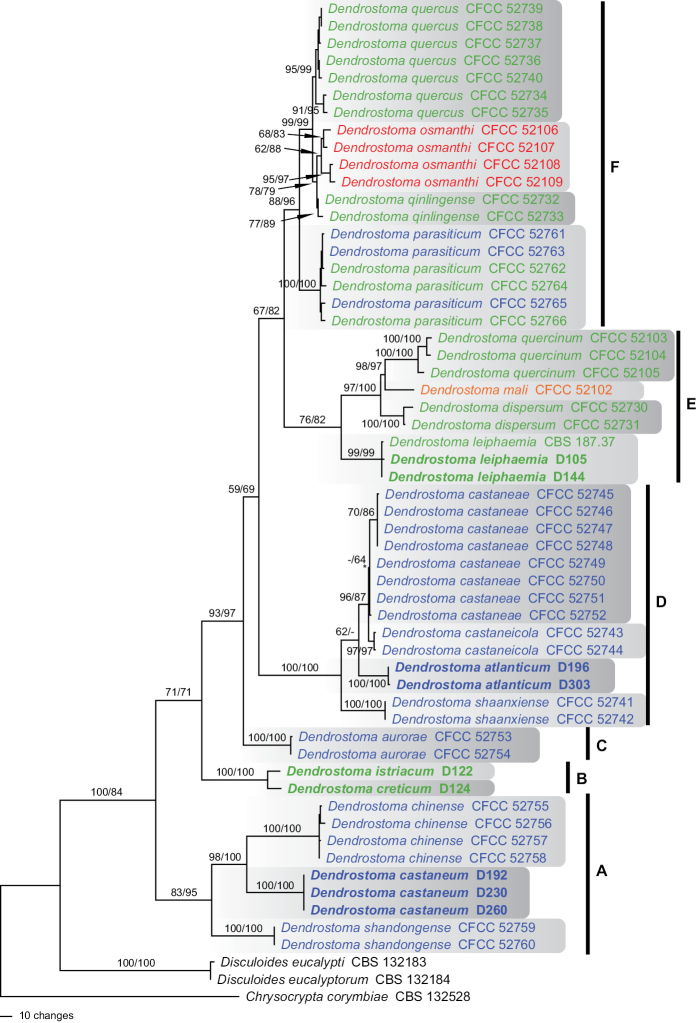
Phylogram showing one of 8 MP trees 1552 steps long revealed by PAUP from an analysis of the combined ITS-LSU-*rpb2*-*tef1* matrix of *Dendrostoma*, with *Chrysocrypta corymbiae*, *Disculoides eucalypti* and *D. eucalyptorum* added as outgroup taxa. MP and ML bootstrap support above 50% are given above or below the branches. The asterisk (*) denotes the node collapsed in the strict consensus of the eight MP trees. Accessions in bold were sequenced in the present study; accessions in blue were isolated from *Castanea*, those in green from *Quercus*, in orange from *Malus* and in red from *Osmanthus*.

Of the 4194 characters included in the phylogenetic analyses, 703 were parsimony informative (133 from the SSU-ITS-LSU, 247 from *rpb2*, 323 from *tef1*). MP analyses revealed eight MP trees 1552 steps long, one of which is shown as Figure 1. The tree backbone was identical in all MP trees, except for the position of *Dendrostoma castaneicola*, which was embedded within *D. castaneae* in some of the MP trees (not shown). The best ML tree (lnL = −13985.7598) revealed by RAxML was compatible with the MP strict consensus tree, except for an interchanged position of *D. atlanticum* and *D. shaanxiense* (not shown). The genus *Dendrostoma* received maximum and medium support in the MP and ML analyses, respectively, and most of the tree backbone received significant support as well (Fig. 1). Although *Dendrostoma* accessions from *Quercus* and *Castanea* were interspersed, host-related patterns were obvious in the various *Dendrostoma* subclades (Fig. 1). The basal subclade A (*D. castaneum*, *D. chinense*, *D. shandongense*) contains only accessions from *Castanea* and is followed by subclade B (*D. creticum*, *D. istriacum*) from *Quercus* and subclade C with the single species *D. aurorae* from *Castanea*. Subclade D contains four species from *Castanea* (*D. atlanticum*, *D. castaneae*, *D. castaneicola*, *D. shaanxiense*) and subclade E three species from *Quercus* (*D. dispersum*, *D. leiphaemia*, *D. quercinum*) plus *D. mali* from *Malus* (Rosaceae). Finally, subclade F contains *D. qinlingense* and *D. quercus* from *Quercus*, *D. parasiticum* from *Quercus* and *Castanea*, and *D. osmanthi* from *Osmanthus* (Oleaceae). Geographically, no patterns were obvious, as the European accessions were distributed amongst the phylogenetic tree and embedded within lineages described from Eastern Asia (China).

**Figure 2.**
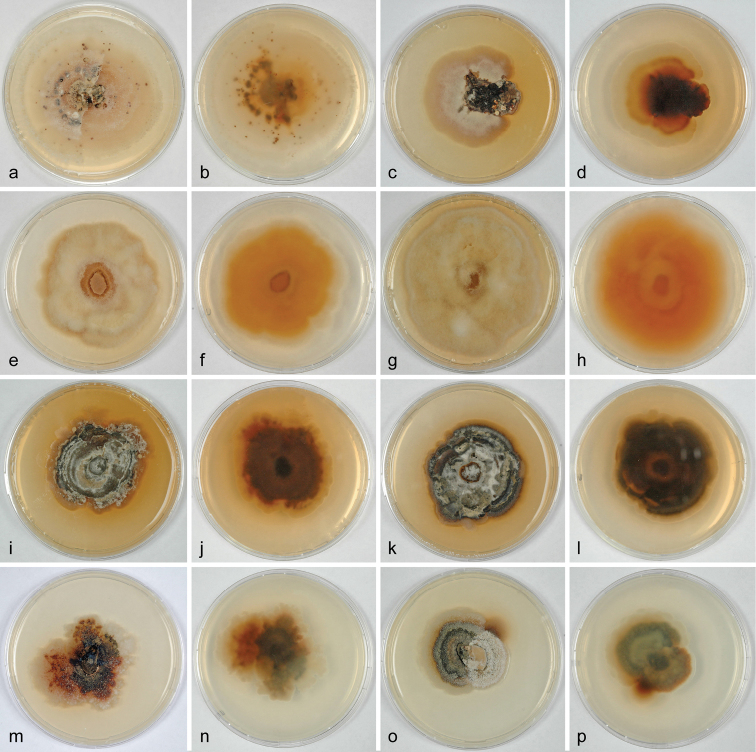
*Dendrostoma* cultures (CMD, 16 °C) after 20d (**e–h, m–p**), 54–58 d (**a–d, i–l**). **a–d***D. atlanticum* (**a, b** D192; **c, d** D230) **e–h***D. castaneum* (**a, b** D196; **c, d** D303) **i–j***D. creticum* D124 **k–l***D. istriacum* D122 **m–p***D. leiphaemia* (**m, n** D105; **o, p** D144) **b, d, f, h, j, l, n, p** reverse side.

### Taxonomy

#### Dendrostoma

Taxon classificationFungiDiaporthalesErythrogloeaceae

X.L. Fan & C.M. Tian, Persoonia 40: 126 (2018)

E946F7CA-DAE8-574E-8ECB-9AE575144053

##### Type species.

*Dendrostoma mali* X.L. Fan & C.M. Tian.

##### Description, emended here.

*Sexual morph*: *pseudostromata* immersed in bark and erumpent, causing a pustulate bark surface, consisting of an ectostromatic disc and entostroma with embedded ascomata. *Ectostromatic disc* flat or convex, surrounded by bark flaps. *Entostroma* light-coloured, prosenchymatous to nearly pseudoparenchymatous, mixed with bark cells, sometimes forming a more-or-less conical central column beneath the disc. *Stromatic zones* lacking or sometimes bark dorsally darkened. *Ascomata* perithecial, subglobose. *Ostioles* flat in the disc or slightly projecting, cylindrical, often with conical apical part. *Paraphyses* deliquescent. *Asci* oblong, fusoid, narrowly clavate or subellipsoid, with a refractive apical ring, containing (4–)6–8 ascospores in various arrangements, becoming detached at maturity. *Ascospores* hyaline, ellipsoid, fusoid, oblong to subacicular, often inequilateral, straight to curved, bicellular, more-or-less constricted at the median or eccentric septum, smooth, with 2–4 drops or multiguttulate, often with gelatinous terminal appendages. *Asexual morph*: *conidiomata* acervular, either forming lateral locules on the ostiolar level of sexual pseudostromata or separate, conical to pulvinate, immersed-erumpent from bark; wall pseudoparenchymatous. Often a pseudoparenchymatous conical central column present beneath the covering layer. *Conidiophores* non-differentiated, hypha-like or reduced to conidiogenous cells. *Conidiogenous cells* phialidic, lining the inner walls of cavities, subcylindrical to ampulliform, hyaline, shades of brown with age. *Conidia* hyaline, aseptate, smooth, multiguttulate or not, thin-walled, oblong, ellipsoid to fusoid, straight or curved.

#### Dendrostoma atlanticum

Taxon classificationFungiDiaporthalesErythrogloeaceae

Voglmayr & Jaklitsch sp. nov.

85B0A4DA-CE77-5FFC-B208-521D1DABACF8

832515

Figures 3 , 4

##### Diagnosis.

*Dendrostoma atlanticum* is recognized by clay-coloured ectostromatic discs and ascospores having large guttules and bristle-like appendages.

##### Holotype.

France, Bretagne, Dépt. Morbihan (56), Saint Martin sur Oust, Beauvais, on twigs of *Castanea sativa*, soc. immature *Valsaria* sp., 15 Jan. 2016, A. Delannoy (WU 37024; ex-type culture CBS 145804 = D196).

##### Etymology.

*Atlanticum*, referring to its occurrence in the Atlantic region.

##### Description.

*Sexual morph*: *pseudostromata* 1–4.5 mm in their widest dimension in cross section, bluntly conical or pulvinate, circular, elliptic or irregular in outline, scattered, gregarious to confluent up to 7 mm length. *Ectostromatic discs* 0.4–2 mm in their widest dimension, distinct and conspicuous, projecting up to 0.5(–1) mm from the bark surface, pulvinate, circular, angular or fusoid in outline, with flat or convex top, initially whitish, turning pale to dark clay-coloured, splitting the periderm, often surrounded by bark flaps. *Ostioles* 1–40 per disc, often originating eccentrically from the perithecial venter, arranged in ring-like configuration or variably filling the disc, (44–)100–163(–195) µm (*n* = 42) in diameter at the tip, brown to black, cylindrical, sometimes attenuated towards tip, plane with the disc or projecting up to 300 µm; tip usually with dark umbilicate centre. *Entostroma* whitish, yellowish to pale bark coloured, consisting of thin-walled, hyaline to subhyaline 1–3 µm wide hyphae and bark cells. *Perithecia* (390–)480–660(–750) µm (*n* = 35) in diameter, depressed subglobose, collapsing upward upon drying; *peridium* ca 10–30 µm thick, colourless to pale olivaceous, consisting of hyaline to yellowish or pale brownish, thick-walled cells without clear contours, smaller and more-or-less isodiametric outside, larger and compressed inside, very variable, (3–)4–17(–38) in diameter (*n* = 66). *Paraphyses* of broad collapsing threads. *Asci* (64–)71–86(–90) × (11–)13–17(–19) µm (*n* = 35), fusoid to oblong, being released at maturity, containing 8 biseriate ascospores. *Ascospores* (13–)15–18(–20) × (4.3–)5.5–7(–8) µm, l/w (2.1–)2.4–2.9(–3.9) (*n* = 51), ellipsoid, often inequilateral, 2-celled, slightly constricted at the median septum, with the upper cell often slightly wider than the lower, hyaline, with 1–2 large and several small guttules per cell, smooth, with a hyaline, bristle-like, straight to curved appendage (10–)11.5–15.5(–21) × (1.5–)2–2.5(–2.8) µm (*n* = 101) at each end.

*Asexual morph* acervular. *Conidiomata* ca 1–2.2 mm in diameter, bluntly conical, width exceeding height, prosenchymatous. *Covering discs* 0.3–1.1 mm in diameter, flat to pulvinate, whitish cream to pale reddish brown. Central column whitish to reddish brown, usually darker toward the top; fertile chamber ring-like around the central column; walls and column consisting of pale yellowish brown *textura angularis*, outer wall and outer layer of the column containing numerous crystals. *Phialides* (3.7–)6.3–9.7(–11.5) × (2–)2.5–3.8(–4.7) µm (*n* = 46), arranged in a palisade on hyaline to yellowish, angular cells, ampulliform to lageniform, less commonly cylindrical. *Conidia* 1-celled, hyaline, smooth, dimorphic, both morphs formed in the same locule, either ellipsoid to oblong, (6.4–)7.7–10.2(–11.7) × (4–)4.5–5.7(–6) µm, l/w (1.4–)1.4–2.2(–3) (*n* = 21), with a large guttule and often distinct abscission scar, or cylindrical, (7.7–)10.2–13.5(–15.3) × (2.3–)2.5–3.2(–3.5) µm, l/w (2.8–)3.6–4.7(–5.6) (n = 45), straight or curved, with mostly 3 or 4 confluent guttules.

##### Culture characteristics.

On CMD at 16 °C in the dark colony more-or-less circular, of loose mycelium, first white, variably covered by white aerial hyphae, becoming dense, forming white and apricot to orange zones, darkening and turning black from the centre, sometimes forming reddish brown dots, spots or tubercles.

##### Other specimen examined.

Spain, Galicia, Pontevedra, O Grove, 42°28'04"N, 08°53'14"W, on twigs of *Castanea sativa*, 4 Nov. 2018, M.A. Delgado (WU 37025; culture D303).

##### Notes.

*Dendrostoma atlanticum* is easily recognized by its long-pedicellate ascospores having 2–4 large drops, setting it apart from *D. castaneum*, which has narrow, often curved ascospores with small drops and short appendages. All species described from *Castanea* in China are only known from asexual morphs (Jiang et al. 2019).

**Figure 3.**
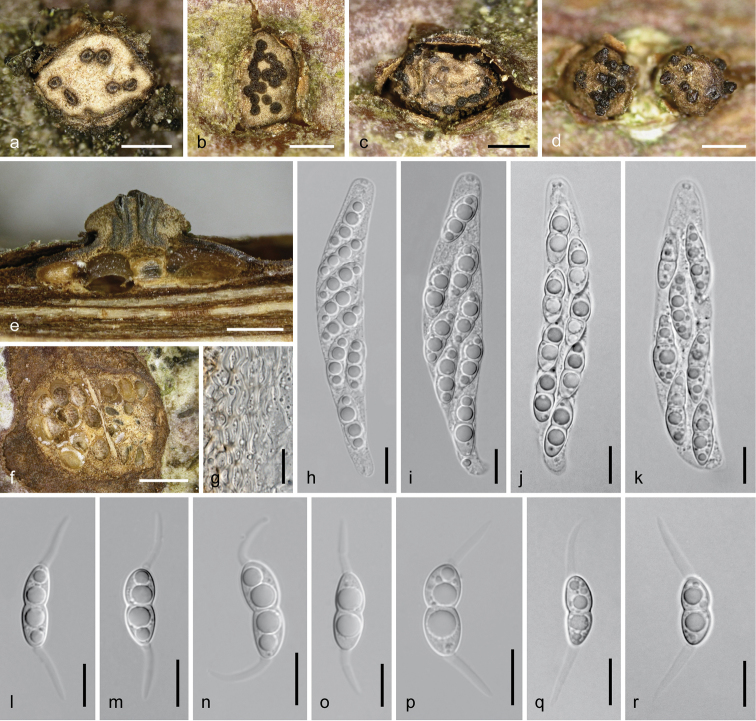
*Dendrostoma atlanticum*. Sexual morph **a–d** ectostromatic discs and ostioles **e** pseudostroma in vertical section **f** pseudostroma in cross section **g** peridium in cross section (in 3% KOH) **h–k** asci **l–r** ascospores. **a–c, f, h, i, k–p**WU 37024 = D196), **d, e, g, j, q, r**WU 37025 = D303. Scale bars: 1 mm (**f**), 500 µm (**a–e**), 10 µm (**g–r**).

**Figure 4.**
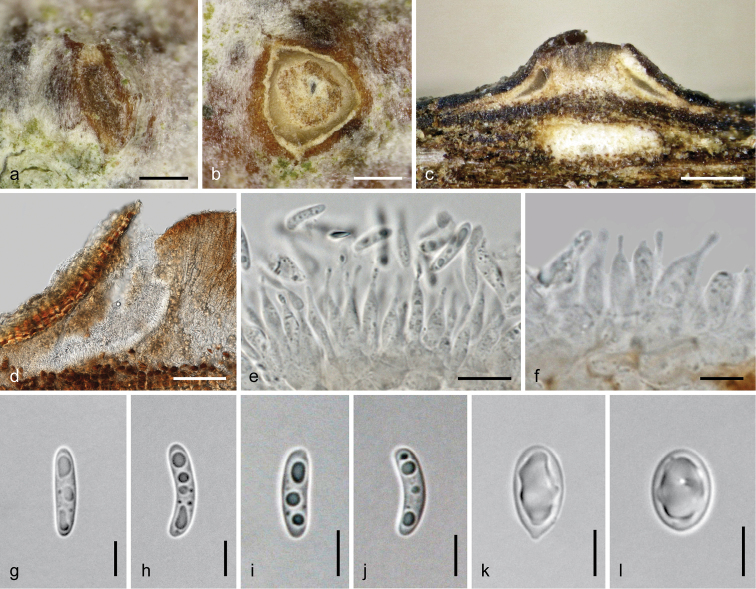
*Dendrostoma atlanticum* (WU 37024 = D196). Asexual morph **a, b** conidiomata in face view **c** conidioma in vertical section **d** vertical section through fertile chamber and part of the central column **e, f** phialides **g–l** conidia (cylindrical in **g–j**, ellipsoid in **k, l**). **d–l** In 3% KOH. Scale bars: 300 µm (**a–c**), 100 µm (**d**), 10 µm (**e**), 5 µm (**f–l**).

#### Dendrostoma castaneum

Taxon classificationFungiDiaporthalesErythrogloeaceae

(Tul. & C. Tul.) Voglmayr & Jaklitsch comb. nov.

663BB86E-989B-53AC-B393-21579EE9F1D8

832516

Figures 5 , 6

Valsa castanea Tul. & C. Tul., Select. fung. carpol. (Paris) 2: 202 (1863) (Basionym).
≡ Amphiporthe castanea (Tul. & C. Tul.) M.E. Barr, Mycol. Mem. 7: 142 (1978).
≡ Cryptodiaporthe castanea (Tul. & C. Tul.) Wehm., Trans. Br. mycol. Soc. 18(4): 284 (1934) [1933].
≡ Diaporthe castanea (Tul. & C. Tul.) Sacc., Syll. fung. (Abellini) 1: 624 (1882).
= Cryptospora leiphaemoides Fuckel, Jb. nassau. Ver. Naturk. 25–26: 323 (1871).
≡ Diaporthe leiphaemoides (Fuckel) Sacc., Syll. fung. (Abellini) 1: 624 (1882).

##### Diagnosis.

*Dendrostoma castaneum* is recognized by KOH+ purple ostioles, slender ascospores with small drops and subfusiform conidia, and the presence of hyphal conidiophores.

##### Description.

*Sexual morph*: *pseudostromata* 0.8–3(–5) mm in their widest dimension in cross section, very variable, flat subconical or lenticular, in outline circular, elliptic or elongate, scattered, gregarious or confluent, and forming elongate patches, lifting the periderm slightly and often becoming visible as a dark zone on the bark surface, causing bumps in bark, splitting the periderm. *Ectostromatic discs* 0.3–2.7 mm in their widest dimension, often ill-defined and variable, cream, yellowish brown to dark brown, flat, surrounded by bark flaps, first present as a covering layer with ostiolar necks subsequently bursting through it, soon crumbling away. *Ostioles* 1–25 per disc, usually arising eccentrically from the perithecial venter, (53–)71–125(–180) µm (*n* = 51) in diameter, bluntly conical or cylindrical with black sides and red, yellowish, or greenish tip, often attenuated to a minute, ca 20–40 µm wide dark centre, in section rounded to angular, sometimes sulcate, variably arranged in the disc, projecting to 0.2 mm, periphysate; red colour of the ostiolar tip turning purple in 3% KOH and yellow in lactic acid. *Entostroma* yellowish to shades of brown, consisting of bark cells and hyaline to yellowish, 1.5–4.5 wide, thin-walled hyphae becoming thicker-walled and forming a pseudoparenchyma in the vicinity of perithecia. *Perithecia* tightly aggregated, (265–)305–460(–600) µm (*n* = 47) in diameter, depressed subglobose to ellipsoid, collapsing upward; *peridium* ca 10–30 µm thick, hyaline, pale olivaceous to brown, in section outside of brown isodiametric to strongly compressed thick-walled cells, inside of compressed and elongated hyaline to brownish cells, in combination (3–)4–15(–28) µm (*n* = 57) in diameter. *Paraphyses* absent at maturity. *Asci* (49–)53–63(–65) × (7.8–)8.5–10.5(–12) µm (*n* = 35), narrowly clavate to subfusoid or oblong, floating freely in the centre, thick-walled at the apex containing a minute refractive ring invisible in 3% KOH, containing 4–8 biseriate ascospores. *Ascospores* (11.5–)14–18(–20) × (3–)3.5–4.5(–5.3) µm, l/w (2.7–)3.5–4.6(–5.4) (*n* = 76), 2-celled, not or slightly constricted at the median or slightly eccentric septum, oblong to inequilaterally ellipsoid, straight to mostly curved, with the upper cell often slightly wider than the lower, broadly rounded at the ends, hyaline, with several minute drops (confluent to 2 larger drops per cell in mounts), smooth, with or without a hyaline, subconical to filiform appendage (2.2–)2.8–4.5(–5.5) × (1.1–)1.3–1.6(–1.8) µm (*n* = 88) at each end invisible in 3% KOH.

*Asexual morph* co-occurring with the sexual morph, acervular, pulvinate, scattered to aggregated, 0.5–2.7 mm in diameter, appearing as superficial discs 0.3–2 mm in diameter, with undulate surface, cream to pale brown and becoming brittle in the centre and nearly black at the periphery and often also indicated as dark zone on the bark surface around the disc; inside consisting of a pale or yellowish brown, loose and brittle central column consisting of pale brown *t. prismatica* and a lateral ring-like, dense, white to distinctly yellow fertile part with even or undulating margin, the latter also raising above the column, outside surrounded by a partly undulating, ca 20–25 µm thick black wall consisting of dark brown *textura angularis* of cells 4–10 µm in diameter at apical and upper peripheral regions, becoming paler downward and being absent at the base and lower sides. Interior of the fertile chambers consisting of isodiametric to elongate hyaline supporting cells and richly and irregularly branched hyphal conidiophores bearing phialides and conidia. Wall, supporting cells and phialides turning dilute violaceous in 3% KOH. *Phialides* arranged on supporting cells in palisades along the walls and on conidiophores, (6–)8.2–12(–15.3) × (1.7–)2.5–3.5(–5) µm (*n* = 80), repetitive, mostly lageniform, often with long necks; conidia also formed on cylindrical pegs and denticles. *Conidia* (6–)6.7–8(–8.8) × (2.5–)3–3.5(–3.7) µm, l/w (1.7–)2.1–2.6(–3.1) (*n* = 85), subfusiform, subclavate or ellipsoid, scar often distinct, smooth, with few minute drops.

##### Culture characteristics.

On CMD at 16 °C in the dark colony circular, dense, white, covered by white cottony aerial hyphae, partly turning pale apricot, reverse orange, not zonate.

##### Specimens examined

(all on recently detached twigs of *Castanea sativa* on ground). AUSTRIA, Burgenland, Forchtenstein, Kohlstatt, 13 Feb. 2016, H. Voglmayr (WU 37026); Steiermark, near highway A2 exit Steinberg, grid square 9057/1, 26 Oct. 2000, W. Jaklitsch W.J. 1651 (WU 37027); same locality, soc. *Cytospora* sp., 3 Nov. 2015, W. Jaklitsch & H. Voglmayr (WU 37028; culture CBS 145803 = D192). ITALY, Sicilia, Etna, above Zafferana Etnea, soc. *Cytospora* sp. (*Valsa* morph), 17 June 2016, H. Voglmayr & W. Jaklitsch (WU 37029; culture D260); Veneto, Selva di Montello, 8 Apr. 2016, H. Voglmayr & W. Jaklitsch (WU 37030; culture D230).

##### Notes.

Sizes of pseudostromata and acervuli strongly depend on twig thickness. Remarkably, red colour of the ostiolar tip, when present, turns purple in 3% KOH and yellow in lactic acid, a feature, which is typical of the Hypocreales and within the Diaporthales otherwise only found in the Cryphonectriaceae.

So far, confirmed records of *D. castaneum* are only known from Europe where the species is widely co-occurring with its host, *Castanea sativa*. Kobayashi (1970) reported and illustrated *D. castaneum* (as *Cryptodiaporthe castanea*) from *Castanea crenata* and *C. mollissima* in Japan. However, it is unlikely that these collections are conspecific with the European *D. castaneum*, considering their different spore shape and hosts. The 1 or 2 large guttules per ascospore cell and the ascospore appendages illustrated in Kobayashi (1970: fig. 32) are similar to *D. atlanticum* rather than to *D. castaneum*. Remarkably, he also reported and illustrated dimorphic conidia for the Japanese collections, which we also observed in *D. atlanticum*. Considering hosts and distribution, the Japanese collections likely represent one of the species described by Jiang et al. (2019) or an undescribed species.

**Figure 5.**
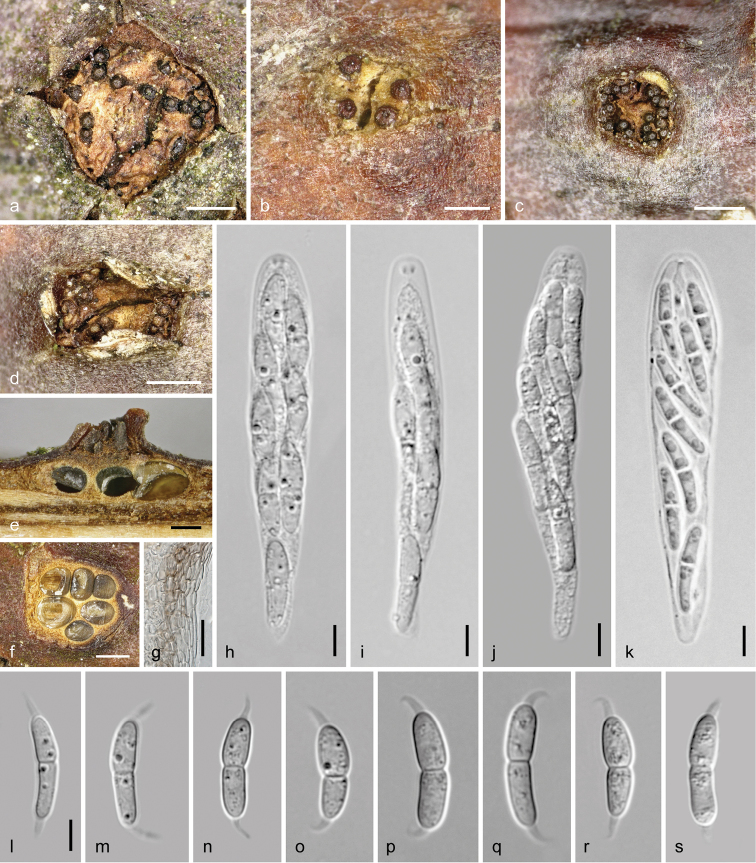
*Dendrostoma castaneum*. Sexual morph **a–d** ectostromatic discs and ostioles (in a ostioles breaking through covering layer) **e** pseudostroma in vertical section **f** pseudostroma in cross section **g** peridium in section (in 3% KOH) **h–k** asci **l–s** ascospores **a, c–g, j, k, s**WU 37030 = D230 **b, n–r**WU 37026 **h, i, l, m**WU 37028 = D192. Scale bars: 500 mm (**a, c, d, f**), 200 µm (**b, e**), 20 µm (**g**), 5 µm (**h–s**).

**Figure 6.**
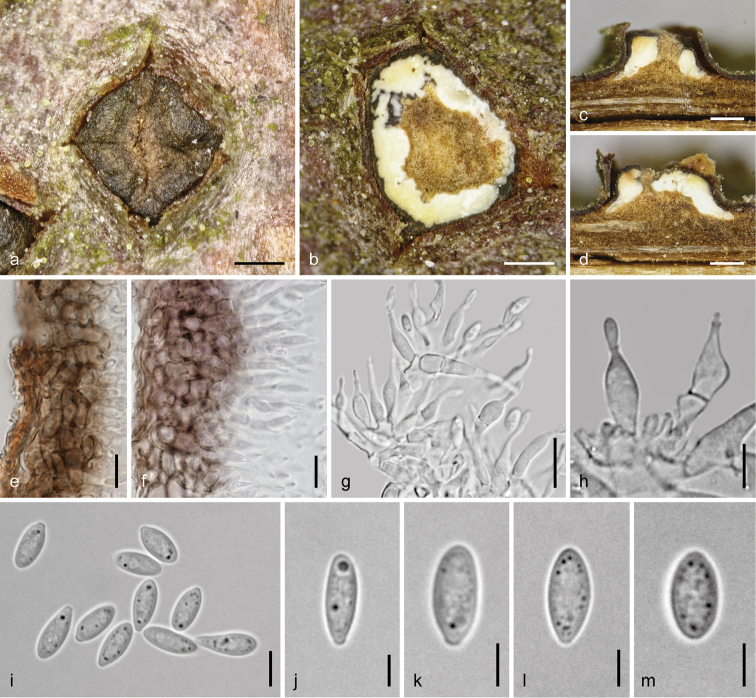
*Dendrostoma castaneum* (WU 37030 = D230). Asexual morph **a** conidioma in face view **b** conidioma in cross section **c, d** conidiomata in vertical section **e** outer upper wall of fertile chamber **f** wall, short conidiophores and phialides (note violaceous tone) **g, h** phialides and hyphal conidiophores **i–m** conidia **f–m** In 3% KOH. Scale bars: 300 µm (**a–d**), 10 µm (**e–g**), 5 µm (**h, i**), 3 µm (**j–m**).

#### Dendrostoma creticum

Taxon classificationFungiDiaporthalesErythrogloeaceae

Voglmayr & Jaklitsch sp. nov.

FF6D3DB6-1E70-5629-88FC-893E4AC1FE15

832517

Figure 7

##### Diagnosis.

*Dendrostoma creticum* is recognized by long, subacicular ascospores.

##### Holotype.

Greece, Crete, near Askifou, 35°17'47"N, 24°12'33"E, on twigs of *Quercus coccifera*, soc. *Cytospora* (*Valsa* morph) sp., 6 June 2015, H. Voglmayr & W. Jaklitsch (WU 37031; ex-type culture CBS 145802 = D124)

##### Etymology.

*Creticum*, referring to its occurrence, Crete.

##### Description.

*Sexual morph*: *pseudostromata* 0.6–1.6 mm in their widest dimension in cross section, pulvinate, circular, elliptic or irregular in outline, scattered, gregarious to confluent up to 4 mm length, causing small bumps in the bark, splitting the periderm. *Ectostromatic discs* 0.25–1.4 mm in their widest dimension, medium to dark brown, flat or convex, surrounded by bark flaps. *Ostioles* 1–7 per disc, (31–)55–102(–135) µm (*n* = 40) in diameter at the rounded tip, dark brown to black, bluntly conical, plane with the disc or slightly prominent. *Entostroma* pale bark coloured, mottled. *Perithecia* (245–)320–445(–495) µm (*n* = 30) in diameter, depressed-subglobose, collapsing upward; *peridium* ca 10–50 µm thick, a dark brown *textura angularis* in face view, in section outside of dark brown *textura angularis* to strongly compressed cells (4–)7–14(–18) µm (*n* = 30) in diameter, inside of strongly compressed and elongated hyaline cells. *Paraphyses* absent at maturity. *Asci* (66–)71–85(–94) × (8.8–)9.5–11.2(–12.3) µm (*n* = 44), narrowly clavate to subfusoid, floating freely in the centre, containing 8 bi- to triseriate ascospores. *Ascospores* (26–)33–45.5(–52) × (2.7–)3–3.7(–4.6) µm, l/w (6.8–)9.8–14.3(–17.5) (n = 40), 2-celled, slightly constricted at the median or often distinctly eccentric septum, oblong, straight to curved, with the upper cell often slightly wider than the lower, hyaline, multiguttulate, smooth, with or without a hyaline subconical appendage (1.4–)1.5–2.3(–3.2) × (0.6–)0.9–1.3(–1.5) µm (*n* = 25) at each end.

*Asexual morph* unknown.

##### Culture characteristics.

On CMD at 16 °C in the dark colony circular to irregular, dense, white, partly covered by short, white aerial hyphae, zonate, soon turning dark brown to black with pale apricot spots and margin and apricot to orange pigment diffusing into agar, reverse dark brown with orange margin.

##### Notes.

*Dendrostoma creticum* is similar to the closely related *D. istriacum* but differs by distinctly longer ascospores, darker ectostromatic discs and a different host species.

**Figure 7.**
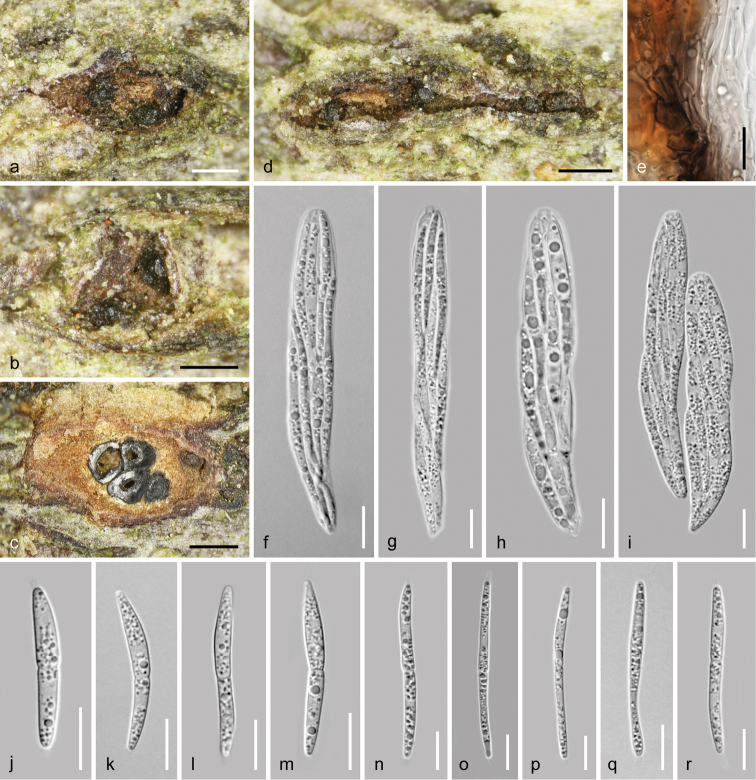
*Dendrostoma creticum* (WU 37031 = D124). **a, b, d** Ectostromatic discs and ostioles in face view **c** pseudostroma in cross section **e** peridium in cross section in 3% KOH **f–i** asci **j–r** ascospores. Scale bars: 200 µm (**a, b, d**), 500 µm (**c**), 10 µm (**e–r**).

#### Dendrostoma istriacum

Taxon classificationFungiDiaporthalesErythrogloeaceae

Voglmayr & Jaklitsch sp. nov.

2B551B72-EB4E-5083-9C57-133A8747AA7A

832518

Figure 8

##### Diagnosis.

*Dendrostoma istriacum* is recognized by narrow, oblong ascospores with small drops.

##### Holotype.

CROATIA, Istria, Rovinj, near Kamp Amarin, 45°06'33"N, 13°37'02"E, on twigs of *Quercus ilex*, soc. *Diplodia* sp., 14 May 2015, H. Voglmayr (WU 37032; ex-type culture CBS 145801 = D122).

##### Etymology.

*Istriacum*, referring to its occurrence, Istria.

##### Description.

*Sexual morph*: *pseudostromata* 0.6–1.5 mm in their widest dimension in cross section, pulvinate, circular or elliptic in outline, scattered or tightly aggregated in large numbers, causing bumps in the bark and bark lesions to ca 3.2 mm long parallel to the twig axis. *Ectostromatic discs* 0.15–0.7 mm in diameter, mostly inconspicuous, surrounded by bark flaps, flat or convex, prosenchymatous, first whitish, turning pale to dark brown, becoming disintegrated and replaced by black ostioles and perithecial tops. *Entostroma* whitish to pale bark coloured. *Stromatic tissues* consisting of bark cells and 2–4 µm wide, hyaline to brown hyphae. *Ostioles* 1–5 per disc, (45–)61–91(–103) µm (*n* = 30) in diameter, short cylindrical, slightly projecting from the disc, brown to black; wall consisting of dark brown *textura angularis*. *Perithecia* (230–)280–393(–443) µm (*n* = 20) in diameter, globose to subglobose; peridium ca 15–35 µm thick, pale olivaceous to dark brown, consisting of 2–4 cell layers of thick-walled, dark brown angular cells (3–)4–13.5(–20.5) µm (*n* = 40) in diameter outside and long compressed, thin-walled, hyaline to brownish cells inside. *Paraphyses* absent at maturity. *Asci* (59–)62–70(–74) × (7–)8.5–10(–11) µm (*n* = 30), fusoid to narrowly clavate, floating freely in the centre, containing 8 bi- to triseriate ascospores. *Ascospores* (19.3–)20.5–25.5(–29.5) × (3–)3.5–4.2(–5.1) µm, l/w (4.5–)5.3–7(–8.7) (*n* = 40), 2-celled, constricted at the more-or-less median septum, oblong, straight to curved, with the upper cell often slightly wider than the lower, hyaline, containing several small guttules concentrated towards the ends and the septum, smooth, with a hyaline subconical appendage (1.7–)2.5–3.5(–4.5) × (0.8–)1–1.3(–1.5) µm (*n* = 40) at each end, becoming elongated in mounts.

*Asexual morph*: *conidiomata* ca 250–520 µm in diameter, acervular, inconspicuous, immersed in bark, causing small bark bumps, becoming visible in fissures, whitish to brownish, flat or convex, bluntly conical, usually broader than high, consisting of a broad sterile greyish brown central column, a white outer fertile ring and a brown covering layer; also fertile between the latter and the top of the column. Covering layer consisting of a dark brown *textura angularis* of 4–10 µm wide cells, turning paler to hyaline and more rounded downwards; column comprising pale brown *textura angularis*-*epidermoidea* of similarly sized cells; outer margin of the fertile ring consisting of a narrow layer of hyaline to pale brown, angular to compressed cells; gel surrounding rounded to angular, subhyaline to hyaline cells supporting phialides slowly turning pinkish in 3% KOH. *Phialides* forming palisades in fertile areas, tightly packed, cylindrical to ampulliform, often with long acute necks, (5.5–)6.3–9(–11) × (1.8–)2.2–3.7(–5.3) µm (*n* = 33). *Conidia* (4–)5–6.6(–7.4) × (1.9–)2.1–2.5(–2.7) µm, l/w (1.6–)2.1–3(–3.7) (n = 53), oblong to ellipsoid, 1-celled, hyaline, smooth, usually with distinct abscission scar.

##### Culture characteristics.

On CMD at 16 °C in the dark, colony circular to irregular, dense, white, partly covered by short white aerial hyphae, zonate, soon turning dark brown to black with pale apricot to reddish brown spots and margin and some pale apricot pigment diffusing into agar, reverse dark brown with pale apricot margin.

##### Other specimen examined.

CROATIA, Istria, Rovinj, near Kamp Veštar, 45°03'19"N, 13°40'55"E, on twigs of *Quercus ilex*, 30 May 2019, H. Voglmayr (WU 37033).

##### Notes.

*Dendrostoma istriacum* is closely related to *D. creticum* but differs from that species by distinctly shorter ascospores and a different host species.

**Figure 8.**
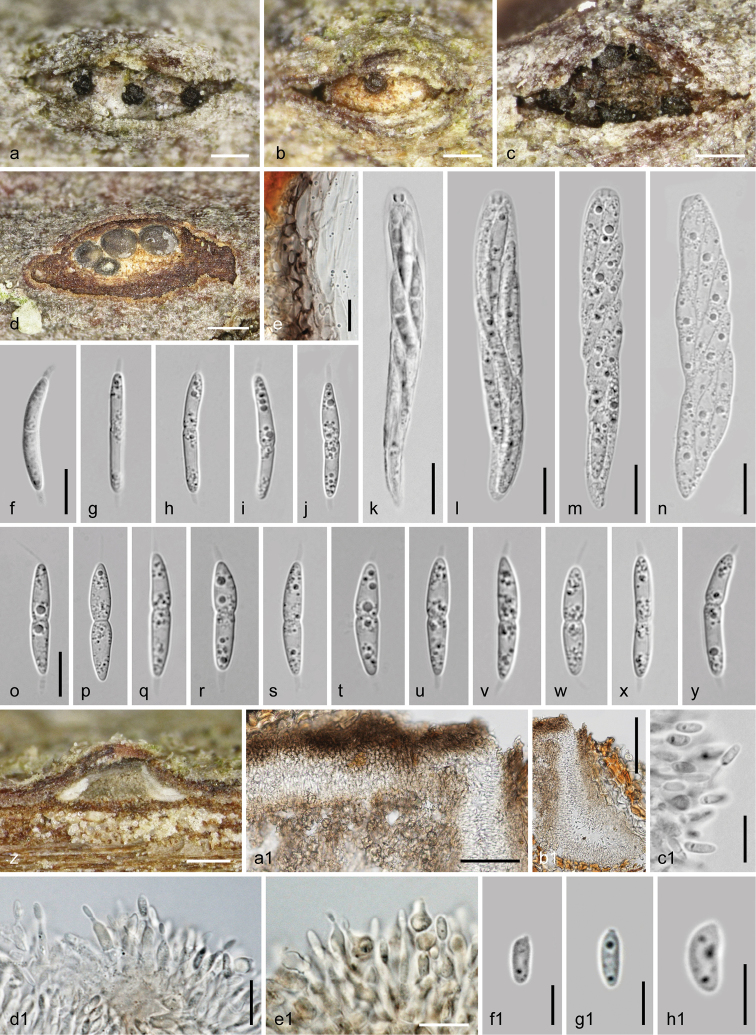
*Dendrostoma istriacum* (WU 37032 = D122). **a–y** Sexual morph **a–c** ectostromatic discs and ostioles **d** pseudostroma in cross section **e** peridium in cross section **f–j, o–y** ascospores **k–n** asci **z–h1** asexual morph **z** conidioma in vertical section **a1** upper part of conidioma showing covering layer, upper part of central column and fertile layers with opening at the upper right side **b1** peripheral fertile chamber in vertical section **c1** conidia attached to phialides **d1–e1** phialides **f1–h1** conidia; **e, c1–h1** In 3% KOH. Scale bars: 150 µm (**a–c, z**), 300 µm (**d**), 100 µm (**b1**), 50 µm (**a1**), 10 µm (**e–y, c1–e1**), 5 µm (**f1–h1**).

#### Dendrostoma leiphaemia

Taxon classificationFungiDiaporthalesErythrogloeaceae

(Fr.: Fr.) Senan. & K.D. Hyde, in Senanayake et al., Fungal Diversity 93: 317 (2018).

52FCF040-403F-501A-896D-8AB2B45ED82B

Figure 9

Sphaeria leiphaemia Fr., Syst. mycol. (Lundae) 2(2): 399 (1823) (Basionym).
≡ Amphiporthe leiphaemia (Fr.) Butin, Sydowia 33: 22 (1980).
≡ Diaporthe leiphaemia (Fr. : Fr.) Sacc. [as ‘leiphaema’], Atti Soc. Veneto-Trent. Sci. Nat. 2(1): 135 (1873).
≡ Valsa leiphaemia (Fr.) Fr., Summa veg. Scand., Sectio Post. (Stockholm): 412 (1849).

##### Diagnosis.

*Dendrostoma leiphaemia* is recognized by conspicuous ectostromatic discs, broad conical ostiolar necks, and broad multiguttulate ascospores.

##### Description.

*Sexual morph*: *pseudostromata* 1–5 mm in their widest dimension in cross section, pulvinate to conical, circular, elliptic or irregular in outline, scattered, aggregated to confluent, sometimes forming lines of up to 15 mm length, causing conspicuous bumps and lesions in the bark; dark brown dorsal zones present within the bark, absent in basal regions. *Ectostromatic discs* 0.35–2.5 mm in their widest dimension, conspicuous, whitish, cream, pale brown, pale yellowish brown to dull brown, fusoid, triangular to circular in section, flat or convex, often surrounded by bark flaps, elevated up to 1.3 mm beyond the bark surface, brittle to powdery, first present as a covering layer with ostiolar necks subsequently bursting through it, eventually crumbling away. *Ostioles* 1–30 per disc, (88–)124–220(–336) µm (*n* = 64) in diameter, dark brown, black, or reddish brown with black, rarely yellowish tip, cylindrical with conical apical part, attenuated to 35–90(–180) µm at the rounded, compressed or coarsely sulcate tip, projecting to 250, less commonly 400 µm, white, in upper regions sometimes yellow inside, periphysate, arising centrally to eccentrically from the perithecial venter and slightly convergent above perithecia; turning partly yellow, partly brown in 3% KOH. *Entostroma* whitish to pale yellowish or pale bark-coloured, prosenchymatous to pseudoparenchymatous, the latter particularly in the vicinity of perithecia, consisting of 1.5–5 µm wide hyphae or angular cells, mixed with bark cells. *Perithecia* arranged in valsoid configuration, tightly aggregated, (292–)380–625(–700) µm (*n* = 21) in diameter, globose to depressed-subglobose, with gelatinous contents, collapsing upward; *peridium* ca 7–35 µm thick, pale olivaceous to dark brown, consisting of an outer layer of isodiametric to elongate, thick-walled dark brown cells and an inner layer of compressed elongate, hyaline to brownish, thin-walled cells (5–)6.5–16(–22.5) µm (*n* = 31). *Paraphyses* absent at maturity. *Asci* floating freely in the centre when mature, (49–)58–71(–80) × (9–)10–13.5(–17.5) µm (*n* = 56), clavate, oblong, fusoid to subellipsoid, with a refractive apical ring, containing 8 bi- to triseriate, fasciculate or obliquely uniseriate ascospores. *Ascospores* (15–)16–19(–21) × (3.8–)4.3–5.2(–5.8) µm, l/w (2.7–)3.3–4.1(–4.7) (*n* = 95), 2-celled, not or slightly constricted at the median or slightly eccentric septum, inequilaterally ellipsoid or oblong, straight or curved, with the upper cell sometimes slightly wider than the lower, hyaline, multiguttulate, smooth, lacking appendages.

*Asexual morph* co-occurring with the sexual morph, acervular, either present as locules in lateral regions of pseudostromata above perithecia or forming separate, conical to pulvinate, dorsally blackened *acervuli* 0.9–2.2 mm in diameter, with conical upper part or whitish to cream or brownish, more-or-less circular, continous or deeply fissured discs ca 0.4–1 mm in diameter and whitish-cream, partly hollow interior containing slightly darker fertile chambers meandering through it. Walls and interior consisting of brown or hyaline to pale yellowish brown *textura angularis*. *Phialides* lining inner wall of the cavity, sessile, (4.8–)6.5–11(–12.7) × (1.7–)2–3.8(–5.3) µm (*n* = 16), subcylindrical to lageniform, reddish brown in 3% KOH (when old). *Conidia* (4.8–)7–9.5(–11) × (1.5–)1.8–2.3(–2.5) µm, l/w (2.3–)3.3–4.9(–6.3) (*n* = 50), unicellular, cylindrical, oblong, subclavate, rhomboid or narrowly ellipsoid, straight to slightly curved, often with a truncate or acute end, hyaline, turning pinkish-yellowish in 3% KOH, smooth, with minute terminal drops, adhering together in masses when old.

##### Culture characteristics.

On CMD at 16 °C in the dark, colony irregular or dimorphic, dense, white, partly covered by short white aerial hyphae, zonate, soon turning dark brown to black with red or reddish brown spots, reverse dark brown, reddish brown with white, pale apricot or reddish brown spots and margins.

##### Specimens examined.

AUSTRIA, Kärnten, St. Margareten im Rosental, shrubs in front of the Stariwald, grid square 9452/4, on branches of *Quercus petraea*, 9 Jan. 1995, W. Jaklitsch W.J. 443 (WU 37034); same area, 31 Dec. 1997, W. Jaklitsch W.J. 1122 (WU 37035); Niederösterreich, Hagenbrunn, Bisamberg east side, grid square 7664/3, on twigs of *Quercus petraea*, 30 Oct. 1999, W. Jaklitsch W.J. 1396 (WU 37036); Mannersdorf am Leithagebirge, on twigs of *Quercus petraea*, 12 Mar. 2016, H. Voglmayr (specimen lost); Mühlleiten, Herrnau, on branches of *Quercus petraea*, 29 Mar. 2015, H. Voglmayr (WU 37037; culture CBS 145800 = D105); Oberösterreich, Unterach am Attersee, Stockwinkl, Egelsee, grid square 8147/3, on branch of *Quercus petraea*, 25 May 1996, W. Jaklitsch W.J. 880 (WU 37038); Steiermark, Wundschuh, Kaiserwald, at the Seerestaurant, grid square 9058/4, on branch of *Quercus petraea*, 10 Sep. 2002, W. Jaklitsch W.J. 1936 (BPI 843342; culture A.R. 3874); Vienna, 19^th^ district, at the Cobenzl, grid square 7763/2, on branches of *Quercus cerris*, 11 Feb 1995, W. Jaklitsch W.J. 482 (WU 37039); same area and host, 27 Feb. 1999, W. Jaklitsch W.J. 1286 (WU 37040). POLAND, E Grajewo, Kuligi, on branches of *Quercus robur*, 28 July 2015, H. Voglmayr (WU 37041; culture D144).

##### Notes.

Asexual fructifications of this species are reported to have dimorphic conidia (Butin 1980; Wehmeyer 1933). However, for the description above only overmature material with a single type of conidia was available, the measurements of which agree with the cylindrical form given as 7–12 × 1.5–2 µm by Wehmeyer (1933), but their shape is more variable, possibly due to their age. As Butin (1980) observed, the asexual morph precedes the sexual morph and may still be present as separate acervuli among sexual pseudostromata or as locules within the periphery of the latter.

**Figure 9.**
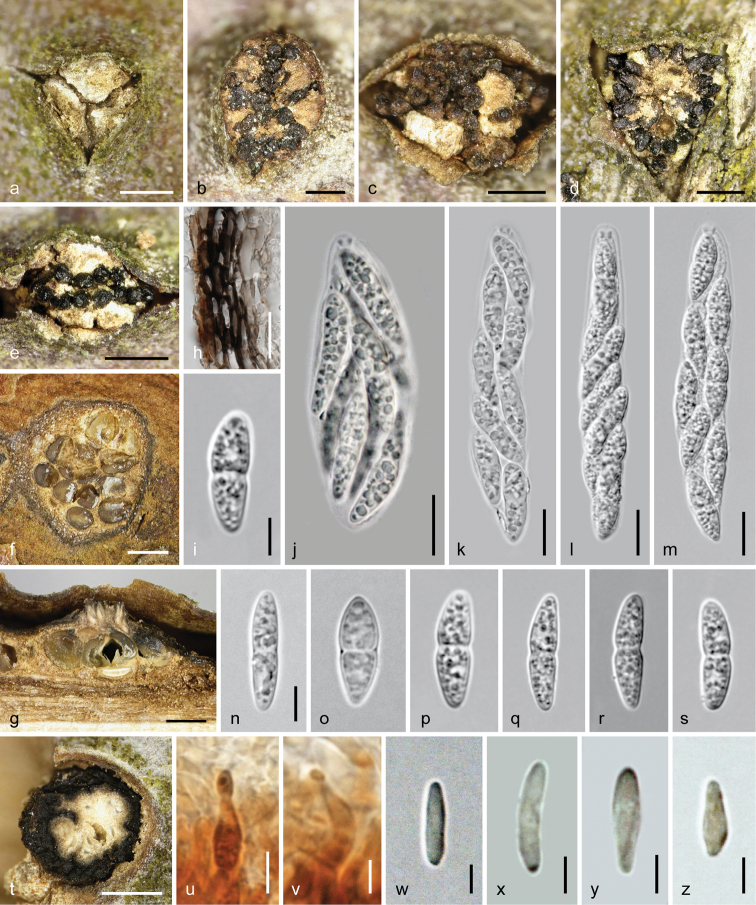
*Dendrostoma leiphaemia*. **a–s** Sexual morph **a–e** ectostromatic discs and ostioles **f** pseudostroma in cross section **g** pseudostroma in vertical section **h** peridium in cross section **i, n–s** ascospores **j–m** asci **t–z** asexual morph **t** conidioma in cross section **u, v** phialides **w–z** conidia; **a, d–g, t–z**WU 37037 (D105), **b**WU 37036 **c, h, j, k, n**WU 37038 **i, l, m, p–s** Mannersdorf **o**WU 37040. **h, j, k, n, o, u–z** In 3% KOH. Scale bars: 500 µm (**a–g, t**), 20 µm (**h**), 10 µm (**j–m**), 5 µm (**i, n–s, u**), 3 µm (**v–z**).

### Key to European species of *Dendrostoma*

**Table:** 

1	Ascospores without appendages, multiguttulate, 15–21 × 4–6 µm; on broad-leaved *Quercus* spp.	***D. leiphaemia***
–	Ascospores with appendages	**2**
2	Appendages 10–21 µm long, bristle-like; ascospores with 1–2 large guttules per cell, 13–19.5 × 4.5–8 µm; on *Castanea sativa*	*** D. atlanticum***
–	Appendages <6 long, not bristle-like	**3**
3	Ascospores 11.5–20 × 3–5.3 µm, oblong, often curved, with 2 minute guttules per cell; on *Castanea sativa*	***D. castaneum***
–	Ascospores longer	**4**
4	Ascospores oblong, 19–30 × 3–5 µm; on *Quercus ilex*	***D. istriacum***
–	Ascospores oblong to subacicular, 26–52 × 2.7–4.5 µm; on *Quercus coccifera*	***D. creticum***

## Discussion

Our phylogenetic analyses are largely congruent with those of Jiang et al. (2019), and different topological positions of, e.g., *D. aurorae* and *D. parasiticum* concern backbone nodes with low to medium support. A notable difference concerns the position of the generic type, *D. leiphaemia*, which in Jiang et al. (2019) is contained within the *D. osmanthi* – *D. qinlingense* – *D. quercus* clade with medium (80% MP) to high (90% ML) support, while in our analyses it is placed basal to the *D. dispersum* – *D. mali* – *D. quercinum* clade with medium support (76% MP, 88% ML). These differences may be due to different taxon and marker sampling, as in the analyses of Jiang et al. (2019) only the ITS and LSU rDNA were available for *D. leiphaemia*.

Previous authors recorded phytopathogenic potential in all species of *Dendrostoma* studied by them (Fan et al. 2018; Jiang et al. 2019). As an example, *Dendrostoma castaneicola*, *D. castaneae*, and *D. shaanxiense* were reported to cause chestnut canker (termed “Dendrostoma canker”) on *Castanea mollissima* in China (Jiang et al. 2019). It is remarkable that almost all Chinese *Dendrostoma* species recorded as canker pathogens by Jiang et al. (2019) were only found as asexual morphs, which were abundantly produced on the dead twigs. This may, at least partly, be linked to the fact that Jiang et al. (2019) mainly investigated chestnut plantations, in which asexual reproduction of virulent pathotypes may be particularly favoured by genetically uniform host cultivars. However, pathogenicity of these species has not been confirmed by inoculation experiments. Défago (1937) observed canker disease symptoms of *Castanea sativa* after artificial inoculation with *Dendrostoma castaneum*, and Kobayashi (1970) mentioned unpublished inoculation experiments showing pathogenicity of *Dendrostoma* sp. (as *Cryptodiaporthe castanea*) on cultivated Japanese chestnut varieties. Phillips and Burdekin (1982) considered *D. castaneum* to be a weak wound pathogen. In our studies, we have not seen any obvious disease symptoms exhibited by *Castanea* and *Quercus* species infected by species of *Dendrostoma*. The typical habitat of species like *D. castaneum* or *D. leiphaemia* are cut branches piled up on the ground. Species on evergreen *Quercus* spp. may occur on dead branchlets attached to trees, but their appearance is rather inconspicuous, and specific searches are necessary to spot them. However, as our observations have not been conducted to specifically study disease symptoms, it is premature to make predictions about potential pathogenicity, which thus cannot be excluded. Frequent association of *Dendrostoma* spp. with *Cytospora* spp. may suggest weak or facultative parasitism, but inoculation experiments are required to prove pathogenicity by fulfilling Koch’s postulates.

Although other genera of the Erythrogloeaceae produce acervuli, asexual morphs of *Dendrostoma* have been termed pycnidia (Fan et al. 2018; Jiang et al. 2019). This may be due to studies in culture, as asexual fructifications on agar may easily be interpreted as pycnidia, even when no true ostioles are present. However, none of the asexual morphs of the European species we have seen on natural substrates have preformed openings that may be termed ostioles. Therefore, we recognize asexual fructifications of *Dendrostoma* on natural substrates generally as acervuli. Jiang et al. (2019) found dimorphic conidia in a single species of *Dendrostoma*, *D. quercus*. Here we add another such species, *D. atlanticum*. These forms occur at the same time in the same asexual fructifications. However, to gain a complete picture of asexual morphs and elucidate entire life cycles of *Dendrostoma* species, long-term studies may be required, as certain asexual fungi have two different morphs, which may not occur at the same time (Butin 1980).

Most species of *Dendrostoma* are only known as asexual morphs. Only one of the 10 species described by Jiang et al. (2019), *D. quercus*, has a sexual morph. However, it is unclear whether in these species sexual morphs are absent, only rarely produced or have not yet been recorded, e.g., due to unfavourable weather conditions for development, unsuitable substrates or an untimely sampling season. Other species, for which sexual morphs are known are *D. mali* on *Malus spectabilis*, *D. osmanthi* on *Osmanthus fragrans*, and *D. quercinum* on *Quercus acutissima* (Fan et al. 2018). All five species of *Dendrostoma* we describe or redescribe from Europe, two from *Castanea sativa* and three from *Quercus* spp., have sexual morphs and in all but one (*D. creticum*) we found also an asexual morph on the natural hosts.

The high species biodiversity of *Dendrostoma* recorded from Eastern Asia as well as the phylogenetic patterns indicate that the group may have originated in this area. This is also supported by the fact that the European species do not form a monophyletic group, but are embedded within Eastern Asian lineages, indicating that Europe has been colonised from Asia several times independently. In addition, evolutionary radiation may have started on *Castanea* as the basal subclade A exclusively contains accessions from that host (Fig. 1). However, detailed additional studies including other areas as well as hosts are necessary to vigorously test these hypotheses.

## Supplementary Material

XML Treatment for Dendrostoma

XML Treatment for Dendrostoma atlanticum

XML Treatment for Dendrostoma castaneum

XML Treatment for Dendrostoma creticum

XML Treatment for Dendrostoma istriacum

XML Treatment for Dendrostoma leiphaemia
